# Digital Healthcare Applications for Preventing Risk Factors in Dementia: Requirements Engineering in the German Healthcare System

**DOI:** 10.1002/alz.090787

**Published:** 2025-01-09

**Authors:** Marius Gerdes, Markus Schinle, Simon Stock, Willhelm Stork

**Affiliations:** ^1^ Karlsruhe Institute for Technology, Karlsruhe, Baden‐Württemberg Germany; ^2^ Forschungszentrum Informatik FZI, Karlsruhe, Baden‐Württemberg Germany

## Abstract

**Background:**

The increasing prevalence of dementia globally, including Germany, necessitates effective strategies to reduce the burden on patients, caregivers, and healthcare systems. Risk factor prevention plays a crucial role in delaying the onset of severe symptoms and alleviating the strain on resources.

**Method:**

This study focuses on identifying the requirements of a digital healthcare application for risk factor prevention in dementia within the German public healthcare system. A requirements engineering process was employed, involving the identification of user needs and regulatory constraints, the generation of clickable mockups, and user testing with individuals at risk for dementia through semi‐structured interviews (N = 30).

**Result:**

The target group, consisting of individuals at risk for dementia, exhibited a strong interest in using digital healthcare applications. Most demonstrated a high affinity for technology and already utilized digital products in their daily lives. Interventions related to exercise and nutrition were well‐received, while interventions targeting sleep encountered skepticism. The German regulatory framework currently only allows for the development of independent applications, although the preference for a human coach was expressed. However, participants indicated that an AI coach could be a viable alternative in the future, as regulations evolve.

**Conclusion:**

By providing a digital healthcare application for risk factor prevention in dementia, scalability to a wider population can be achieved. This approach bridges the gap between specialized care, which requires significant resources, and the limited resources available in the public healthcare system. The study highlights the potential of digital healthcare applications to increase accessibility and facilitate the integration of risk factor prevention strategies into routine care. Furthermore, it underscores the need for regulatory adaptation to accommodate the evolving landscape of digital healthcare solutions.

